# Chetomin rescues pathogenic phenotype of LRRK2 mutation in drosophila

**DOI:** 10.18632/aging.103843

**Published:** 2020-09-24

**Authors:** Ling Ling Chua, Patrick Ho, Joanne Toh, Eng-King Tan

**Affiliations:** 1 Department of Neurology, National Neuroscience Institute, Singapore General Hospital, Singapore 169856, Singapore; 2 Department of Neurology, National Neuroscience Institute Singapore, Singapore 169857, Singapore; 3 Department of Neurology, Singapore General Hospital Singapore 169856, Singapore; 4 Neuroscience Behavioral Disorders Program, Duke-NUS School, Singapore 169857, Singapore

**Keywords:** drosophila, neurodegeneration, LRRK2, Chetomin

## Abstract

Leucine-rich repeat kinase 2 (LRRK2) is a complex protein kinase involved in a diverse set of functions. Mutations in LRRK2 are a common cause of autosomal dominant familial Parkinson’s disease. Peroxiredoxin 2 (PRDX2) belongs to a family of anti-oxidants that protect cells from oxidative stress. Importantly, PRDX2 is a cytoplasmic protein, similar to Leucine-rich repeat kinase 2, which localizes predominantly in the cytosol. Here, we demonstrated that Leurice-rich repeat kinase 2 phosphorylates PRDX2 in Drosophila, leading to a loss of dopaminergic neurons, climbing ability and shortened lifespan. These pathogenic phenotypes in the LRRK2 Drosophila were rescued with transgenic expression of PRDX2. Chetomin, a PRDX2 mimic, belongs to a class of epidithio-diketopiperazine fungal secondary metabolites (containing a dithiol group that has hydrogen peroxide-reducing activity). As proof of principle, we demonstrated that Chetomin recapitulated the rescue in these mutant Drosophila. Our findings suggest that Chetomin can be a potential therapeutic compound in LRRK2 linked Parkinson’s disease.

## INTRODUCTION

Parkinson’s disease (PD) is a common neurodegenerative disorder that is pathologically characterized by the loss of midbrain dopaminergic (DA) neurons in the substantia nigra and the accumulation of Lewy body aggregate [1]. PD is a multifactorial disease with a complex relationship with genetic and environmental factors.

Leucine-rich repeat kinase 2 (LRRK2) is a large complex protein that consists of armadillo repeats, ankryn-like repeats and leucine-rich repeats at the N-terminal domain, a central catalytic core that contains a GTP-binding Ras of complex (Roc) domain, a carboxy-terminal of Roc (COR) domain and a kinase domain belonging to the serine/threonine kinases, and a WD40 domain at the C-terminus [2, 3]. It is associated with a diverse set of cellular functions including mitochondrial function [4–6], cytoskeletal function [7, 8], autophagy [9, 10] and various signaling pathways [11, 12]. Oxidative stress and neuroinflammation are associated with PD pathophysiology [13, 14] which ultimately leads to dopaminergic neuron (DA) degeneration. Mutations in LRRK2 are the frequent cause of autosomal dominant familial PD and have been shown to have increased susceptibility to oxidative stress. G2019S is one of the most common LRRK2 mutation, affecting 5-6% of familial PD [15, 16]. Studies have shown that G2019S has enhanced kinase activity [17] and this can lead to increased sensitivity to stress [18]. G2019S mutation carriers also showed mitochondrial and autophagy impairments [6, 19] as well as increased alpha synuclein inclusion [20].

Peroxiredoxins (PRDX) are a family of six anti-oxidative proteins that share a common reactive cysteine residue in the N-terminal region and their role to inactivate cellular hydroperoxides [21, 22]. They are classified into 2-Cys, atypical 2-Cys, and 1-Cys Prx subfamilies depending on the number of cys protein and its location [23]. PRDX2 belongs to the 2-Cys family and peroxidase activity of this family is regulated via tyrosine and threonine phosphorylation [24].

The PRDX family protects cells from oxidative stress-induced apoptosis by acting as free radical scavengers and has been associated with neurodegeneration [25, 26]. It helps to regulate the peroxide levels in the cells [27] and removes reactive oxygen species (ROS). On the other side of the scale, excessive peroxidase activity have also been implicated in cell and tissue damage [28]. It can also act as a redox sensor by binding to other proteins and regulating their signaling activities [29, 30]. That is why it is critical to regulate the peroxidase activity to have fine balance between beneficial and adverse effect.

We previously found that mutant LRRK2 enhanced phosphorylation of mitochondrial PRDX3, which led to a suppression of cellular peroxidase activity and aggravated toxicity [31] leading to suppression of LRRK2 mutant phenotype. It highlighted the potential of peroxidases as a neuroprotective vehicle for PD patients with LRRK2 mutations. Per, belonging to the same family, was shown to preserve cognitive function against age-linked hippocampal oxidative damage via signaling pathways involving CREB, CaMKII and ERK [32]. They are also believed to play a role in neuroprotection [33, 34]. Importantly, Peroxiredoxin 2 (PRDX2) is a cytoplasmic protein and is detected in the different neuronal population [35], similar to LRRK2, which localizes predominantly in the cytosol.

Here, we studied the interaction between LRRK2 and PRDX2 and the neuroprotective function of PRDX2 on LRRK2 with *in vitro* and *in vivo* experiments. We showed that LRRK2 interacts with PRDX2 through cell-based studies, and using *Drosophila* as an animal model, we demonstrated that G2019S mutation in LRRK2 increased the phosphorylation of PRDX2. Transgenic expression of PRDX2 and its mimic, Chetomin, were able to rescue the pathogenic phenotype of G2019S in *Drosophila*. Our data provides evidence that PRDX2 confers a neuroprotection on pathogenic LRRK2 and that PRDX2 mimic can be further studied as a potential clinical drug for PD.

## RESULTS

### LRRK2 interacts with PRDX2 *in vitro* and *in vivo*

**Figure 1:**
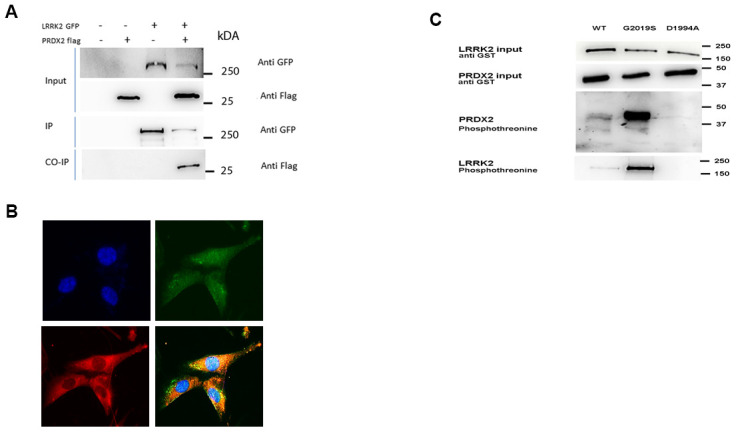
**LRRK2 interacts and phosphorylates PRDX2.** (**A**) LRRK2-GFP and PRDX2-flag are co transfected in HEK293T cells. The lysates were collected and subjected to immunoprecipitation with anti-GFP antibody. The protein was subjected to western blot with anti-flag. Un-transfected cells, LRRK2 GFP alone and PRDX flag alone was transfected into HEK293T cells to serve as negative control. (**B**) Endogenous colocalization of LRRK2 (green) and PRDX2 (red) protein in SKNSH neuronal cells (**C**) *In vitro* kinase assay on SDS-PAGE gel show phosphorylation of PRDX2 by LRRK2 wildtype and G2019S.

We explored the relationship of LRRK2 with PRDX2 *in vitro*, determining if there is a physical interaction between both proteins. The interaction of these two proteins was assessed by co-immunoprecipitation in HEK-293T cells co-transfected with GFP-tagged LRRK2 and FLAG-tagged PRDX2. Following immunoprecipitation of LRRK2 by GFP protein, and immunoblotting for both GFP and FLAG, we found that LRRK2 interacts with PRDX2 *in vitro* (Figure 1A).

We also stained endogenous LRRK2 (Alexa Fluor 488-green) and endogenous PRDX2 (Alexa Fluor 546 dye-red) and showed their colocalization in SKNSH. (Figure 1B) *In vitro* kinase assay was carried out showing that G2019S LRRK2 phosphorylates PRDX2 more than LRRK2 wild-type, with negligible phosphorylation in the presence of kinase dead version of LRRK2 D1994A (Figure 1C).

**Figure 2:**
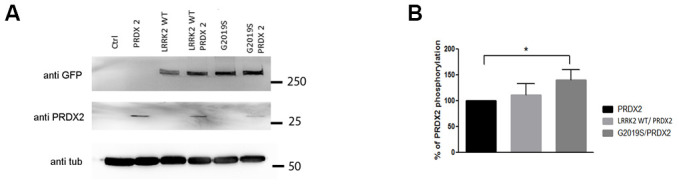
**LRRK2 and PRDX2 transgenic flies.** (**A**) LRRK2 GFP and PRDX2 expression driven by ddc-GAL-4 in transgenic fly head (**B**) Bar graphs show mean percentage and standard deviation of phosphorylation of PRDX2 in LRRK2 wildtype and G2019S flies (n = 3, cohort of 20).

To study their interaction *in vivo*, we generated GFP-tagged *UAS-LRRK2* (wild-type and G2019S) and *UAS-PRDX2* expressing transgenic flies and isogenized them. The *ddc-GAL4* driver were used to drive expression of the various *UAS-LRRK2* transgenes in tyrosine hydroxylase positive (TH+) neurons. Expression levels were verified via immunoblot to ensure that LRRK2 variants and PRDX2 are expressed (Figure 2A). To check for PRDX2 phosphorylation *in vivo*, we used an ELISA assay with a generic Phospho-Threonine/Serine antibody to detect the levels of phosphorylated PRDX2. This method was chosen, as it is more sensitive to detect phosphorylation from the small amount of protein extracted from flies. We found significant percentage increase of PRDX2 phosphorylation in PRDX2 + G2019S double transgenic flies compared to PRDX2 alone (p<0.05). LRRK2 wild-type + PRDX2 showed no substantial increase in phosphorylation (Figure 2B). These results indicated that G2019S mutation in LRRK2 increased the phosphorylation of PRDX2.

### PRDX2 suppress LRRK2 mutant phenotype *in vivo*

**Figure 3:**
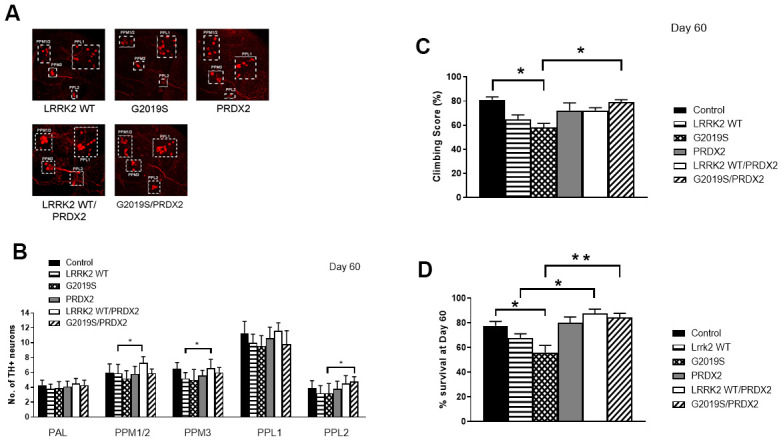
**PRDX2 is able to rescue G2019S pathogenic phenotype.** (**A**) Representative magnified confocal images of whole mount brains 60 days after eclosion. The different clusters of TH+ neurons are boxed up and labeled. (**B**) Bar graphs show number of TH-positive DA neuron clusters in flies at 60 days after eclosion (n = 3, cohort of 10). (**C**) Bar graph shows climbing scores of male flies at 60 days after eclosion. Percentage of flies that reached the top of the column after 1 min was counted (n = 3, cohort of 20). (**D**) Bar graph shows number of flies that survive after 60 days. Age-matched *ddc-GAL4/*+ flies were used as controls. Percentage of flies was tabulated. Significance indicated on the graph: *p<0.05, **p<0.01, ***p<0.001, ****p<0.0001.

**Figure 4:**
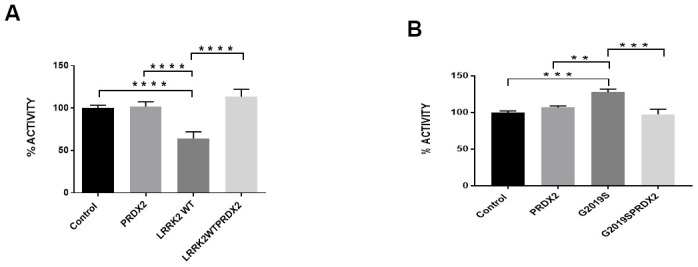
**LRRK2 peroxidase activity.** (**A**) Bar graph show LRRK2 WT peroxidase activity expressed as a percentage normalize to control. (**B**) Bar graph show G2019S peroxidase activity expressed as a percentage normalize to control. Significance indicated on the graph: *p<0.05, **p<0.01, ***p<0.001, ****p<0.0001.

In flies, the expression of PRDX2 was able to partially suppress the loss of TH+ neurons caused by expression of the LRRK2 G2019S protein, as shown by an increase in TH+ neurons in the PPL2 cluster in G2019S + PRDX2 flies (p<0.05). Interestingly, there were also more TH+ neurons in PPM1/2 and PPM3 clusters in wild-type LRRK2 + PRDX2 double transgenic flies compared to wild-type LRRK2 flies (*p <0.05*) (Figure 3A, 3B). At 60 days, G2019S flies displayed significantly lowered climbing ability as compared to control flies. G2019S + PRDX2 flies also displayed a significantly better climbing score compared to G2019S flies (*p <0.05*) after 60 days (Figure 3C). G2019S flies showed reduced lifespan when compared to control flies. The rescue by PRDX2 was also observed in the lifespan assay where aged 60 days old G2019S-PRDX2 and wild-type LRRK2-PRDX2 flies had a significant increase in survival compared to age-matched G2019S and LRRK2 wild-type expressing flies respectively (Figure 3D). These results strongly suggest PRDX2 helps rescue and protect against the pathogenic phenotypes caused by G2019S mutation. LRRK2 wild type flies showed a significant reduction in peroxidase activity when compared to control flies. When co-expressed with PRDX2, there was a significant restoration of peroxidase activity similar to control (*p <0.05*) (Figure 4A). G2019S flies showed an increased peroxidase activity when compared to control flies. The peroxidase activity was restored to normal levels when co-expressed with PRDX2 (Figure 4B). These results suggest that the presence of PRDX2 can help regulate extreme peroxidase activity (either hypo or hyper level) to a basal level.

### Chetomin drug treatment rescues G2019S pathology *in vivo*

Chetomin belongs to a class of epidithio-diketopiperazine (ETP) fungal secondary metabolites that contains a dithiol group that exhibits hydrogen peroxide-reducing activity relying on the thioredoxin system [36]. Thus, as PRDX2 is the only mammalian peroxidase coupled with a thioredoxin/thioredoxin reductase system, Chetomin is a natural compound that can mimic PRDX2 [37, 38]. Hence, we conducted Chetomin drug treatment on G2019S flies to detect if there is a rescue of G2019S pathogenic phenotype.

**Figure 5:**
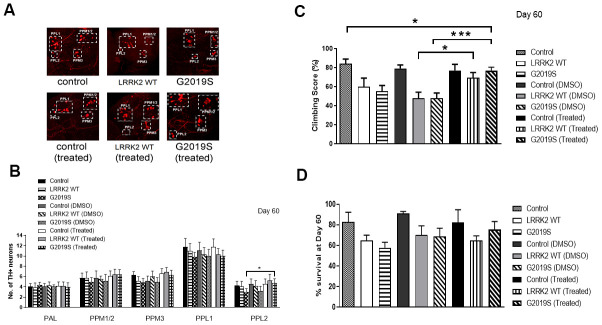
**Challenge by Chetomin rescues G2019S phenotype.** (**A**) Representative magnified confocal images of whole mount brains 60 days after eclosion. The different clusters of TH+ neurons are boxed up and labeled. (**B**) Bar graphs show number of TH-positive DA neuron clusters in flies at 60 days after eclosion (n = 3, cohort of 10). (**C**) Bar graph shows climbing scores of male flies at 60 days after eclosion. Percentage of flies that reached the top of the column after 1 min was counted (n = 3, cohort of 20). (**D**) Bar graph shows number of flies that survive after 60 days. Percentage of flies was tabulated. Significance indicated on the graph: *p<0.05, **p<0.01, ***p<0.001, ****p<0.0001.

Chetomin partially rescued the integrity of DA neurons in PPL2 cluster as we observed a significant increase in the number of TH+ neurons in Chetomin treated G2019S flies compared to without treatment (p<0.05) (Figure 5A, 5B). Chetomin treated G2019S flies fared better in climbing compared to G2019S flies without drug treatment, indicating a rescue of pathogenic phenotype by Chetomin (p<0.05) (Figure 5C). There was also an improvement in the number of flies that survived after 60 days in Chetomin treated G2019S flies compared to G2019S flies without treatment (Figure 5D). The results collectively show that Chetomin is able to rescue the pathogenic phenotype of G2019S LRRK2 mutation in flies.

## DISCUSSION

Mutations in the kinase domain of LRRK2 were shown to increase its kinase activity and worsen the degeneration of DA neurons [18, 39]. These mutations lead to increase susceptibility to oxidative stress, although mechanisms linking both are still unclear. Oxidative stress is the imbalance between free radicals and antioxidants which disrupts key biological processes that leads to cell damage or death. Here, we showed that overexpression of mutant LRRK2 leads to DA neuronal death, decrease climbing ability, shortened lifespan and altered peroxidase activity. The overexpression of transgenic PRDX2 is able to suppress pathogenic LRRK2 phenotypes. We further demonstrated that Chetomin, PRDX2 mimic was able to rescue DA neurodegeneration associated with LRRK2 mutation.

We observed that LRRK2-G2019S kinase mutation causes an increase in the levels of phosphorylated PRDX2. For this mutation, a decreased number of DA neurons and lower climbing ability in the transgenic *Drosophila* were observed. We also observed a higher level of peroxidase activity in G2019S flies but this was restored to a basal level in the presence of PRDX2. Qu et al. showed that PRDX2 binds to Cdk5/p35 and is phosphorylated causing a reduction of PRDX2 peroxidase activity. These led to a downregulation of PRDX2 and increase in oxidative stress that affected DA neuronal loss [40]. Similarly another family of the 2-cys family PRDX1 peroxidase activity was showed to be inactivated by phosphorylation [41]. In G2019S flies there was excessive peroxidase activity and this could contribute to cell and tissue damage [28]. The presence of PRDX2 phosphorylation might serve to regulate peroxidase activity to a normalized level to protect the neurons. This further substantiates the importance of PRDX2 on oxidative stress and its involvement in the PD LRRK2 pathway.

The PRDX family protects cells from oxidative stress-induced apoptosis and has been associated with neurodegeneration [25, 26]. Studies previously have shown that the overexpression of PRDX2 in MN9D neuronal cells led to a decrease in reactive oxygen species [42]. PRDX2 has been shown to preserve cognitive function against hippocampal oxidative damage [32]. It is known that G2019S mutation in LRRK2 enhances oxidative stress-induced neurotoxicity and causes DA neurons to be more susceptible to oxidative stress [43, 44]. Endogenous PRDX2 might be part of this equation as with a lowered expression in LRRK2 G2019S mutation compared to wild-type LRRK2 because it is being phosphorylated, it fails to protect DA neurons from oxidative stress, and thus makes them more vulnerable. When PRDX2 is over expressed, there are more unphosphorylated PRDX2 to provide protection for the DA neurons. This supports our results that showed *in vivo* rescue of G2019S causing phenotypes when PRDX2 is over-expressed.

As proof of principle, challenges with Chetomin, a PRDX2 mimic drug, also recapitulated similar rescue of these phenotypic features in G2019S-mutant expressing flies. Chetomin has been shown to disrupt the interaction between hypoxia-inducible factor-1 (HIF-1) inhibitor by and p300 [37] and also exhibits anti-cancer activities [45, 46]. It was only recently found that the dithiol group in ETP family which Chetomin belongs to, displays hydrogen peroxide-reducing peroxidase activity, and played an effective role in regulating vascular endothelial growth factor receptor-β and vascular endothelial growth factor receptor mediated signaling in vascular cells, promoting healing of vascular injury [38]. Our study is the first to show that Chetomin can be used as a drug to mimic PRDX2 in rescuing the pathogenic phenotypes caused by G201S LRRK2 mutation *in vivo*. This provides the basis to further study the mechanism of Chetomin on PRDX2 and LRRK2.

In conclusion, we provided *in vitro* and *in vivo* evidence of PRDX2 interaction with LRRK2 and phosphorylation of PRDX2 by mutant G2019S LRRK2. We demonstrated that transgenic expression of PRDX2 with LRRK2 ameliorated G2019S induced loss of DA neurons, climbing ability and shortened lifespan. Challenging with Chetomin led to a similar rescue in *Drosophila*. Our findings suggest that chetomin can be a potential therapeutic compound in LRRK2 linked PD. Further studies to validate the effect of PRDX2 on PD patients with LRRK2 kinase mutations and to investigate the neuroprotective effects of Chetomin in other neurodegenerative diseases will be warranted.

## MATERIALS AND METHODS

### Fly stocks

The following flies were used in this study: *dopa decarboxylase* (*ddc*)-GAL4, *yellow white (yw)* (Bloomington *Drosophila* Stock Center). Flies were raised on standard yeast-cornmeal-agar medium at 25ºC with 12-hour light and dark cycle.

### Generation of transgenic strains

Human LRRK2-expressing flies were created by generating transgenic human LRRK2 wild type and variants and point mutations were introduced into LRRK2 using XL Quik change site directed mutagenesis kit and verified by sequencing to ensure the integrity of the cloned ORFs. PRDX2 plasmid (RC207413) was purchased from Origene. LRRK2 wild-type and G2019S cDNA containing a GFP tag at the C-terminus was inserted into the pUAST-attB plasmid, which will allow the UAS constructs to land into a chosen attP site in the fly genome during microinjection. Constructs were sent for microinjection into *Drosophila* embryos (Best Gene).

### Co-immunoprecipitation

HEK293T cells were co-transfected with the plasmids using Turbofect (Thermo Scientific). Cells were collected 24h after transfection for western blot. Transfected HEK293T cells were washed with PBS and lysed in M-PER mammalian protein extraction reagent buffer (Thermo Scientific) supplemented with protease inhibitor and Phos Stop (Roche). The lysates are then incubated with anti GFP conjugated beads (Chemotek) O/N at 4ºC. The precipitates were then washed 5 times using NP40 buffer (50mM Tris pH7.4, 150mM NaCl and 1% NP40) and resuspended in 4X SDS loading buffer.

### Western blot

40 to 50 heads were collected from flies and grinded in M-PER mammalian protein extraction reagent buffer supplemented with protease inhibitor and Phos Stop and placed on ice for 30 mins. They were centrifuged at maximum speed for 15mins and the supernatant was collected for western blot. Protein was extracted from fly head homogenates and equal amounts of protein from the various genotypes were resolved by SDS-PAGE. The following antibodies are used: For LRRK2: anti-GFP (Sigma Aldrich G1544), For PRDX2: anti DYKDDDK/Flag (Cell signaling 2368S) and anti PRDX2 (Sigma Aldrich SAB1406520 and EMD Millipore 07-610)

### Phospho ELISA

PRDX antibody was coated on white 96 wells plate overnight and then blocked in BSA. The fly lysates were collected from 20 fly heads and protein concentration was quantified by thermo-scientific Bradford assay. Equal amount of proteins was added into the plate and incubated with the antibody. After which the plate was washed then it was incubated with generic phosphothreonine/serine HRP (Enzo Lifesciences ADI-KAP-ST2103-E and cell signaling 6949), exposed to chemiluminescence substrate and read by plate reader to detect PRDX2 phosphorylation levels. Background readings from *ddc*/+ flies were substracted from the phospho readings.

### *In vitro* kinase assay

LRRK2 kinase assay was carried out using recombinant truncated LRRK2 protein (Invitrogen PV4873), ATP (Invitrogen PV3227), Kinase buffer (cell signaling) and purified PRDX2-GST protein. The kinase assay was carried out for 2 hours at 30 degrees Celsius. It was stopped by adding 2x SDS loading buffer and boiled for 5 min at 95°C. The protein was loaded on SDS page gel and PRDX2 phosphorylation was detected by phosphor-threonine (cell signaling 9386S).

### Immuno-fluorescence and confocal microscopy

For endogenous cell staining, anti LRRK2 and anti PRDX2 (Sigma Aldrich L9918 and SAB1406520) were used. Flies were aged to day 60 after eclosion, before fly brains were dissected, fixed and stained according to published protocols [26]. Brains were probed with anti GFP and anti-flag for expression immune-florescence. Brains were probed with rabbit anti-tyrosine hydroxylase (1:500, Sigma-Aldrich T2928).

### Climbing and lifespan assays

Climbing assay was performed as described previously [26]. To determine adult lifespan, 100 flies from each genotype under the direction of *ddc-GAL4* were maintained on standard media. Newly eclosed adult flies were transferred into vials containing fresh media every 3 days and mortality was scored at day 60. Age-matched *ddc-GAL4/*+ flies used as controls.

### Peroxidase assay

About 20 fly heads were homogenized lysis buffer and the peroxidase assay was performing according Amplex® Red Hydrogen Peroxide/Peroxidase Assay Kit Catalog no. A22188 from Invitrogen. The results were normalized against the control flies and expressed as a percentage.

### Drug treatment

In drug treated flies, flies were fed with cornmeal-agar medium containing 10uM Chetomin (Sigma), which were first dissolved in DMSO, immediately after eclosion and throughout the entire experimental period.

### Statistical analysis

Quantitative data are expressed as mean ± SEM, unless otherwise stated. Statistical significance for climbing assay, differences in the number of TH-positive DA neurons and peroxidase assay were analyzed using one-way Anova with Bonferroni’s post hoc test, unless otherwise stated. Significance indicated on the graph: *p<0.05, **p<0.01, ***p<0.001, ****p<0.0001.
